# Effects of microtopographic patterns on plant growth and soil improvement in coastal wetlands of the Yellow River Delta

**DOI:** 10.3389/fpls.2023.1162013

**Published:** 2023-03-31

**Authors:** Ke Zhang, Jiangbao Xia, Li Su, Fanglei Gao, Qian Cui, Xianshuang Xing, Mingming Dong, Chuanrong Li

**Affiliations:** ^1^ Shandong Key Laboratory of Eco-Environmental Science for the Yellow River Delta, Binzhou University, Binzhou, Shandong, China; ^2^ College of Forestry, Shandong Agricultural University, Taian, Shandong, China; ^3^ Shandong Hydrology Center, Jinan, Shandong, China

**Keywords:** microtopography, saline soils, soil salinity, soil nutrients, ecological stoichiometry, muddy coastal zones

## Abstract

**Introduction:**

To clarify the effects of microtopography on plant growth and soil water, salt and nutrient characteristics of saline soils in mudflats within muddy coastal zones and explore suitable microtopographic modifications.

**Methods:**

Six microtopographic modification patterns, namely, S-shaped, stripe-shaped, pin-shaped, stepshaped, dense stripe-shaped and crescent-shaped patterns, were established in the coastal mudflats of the Yellow River Delta. The soil water, salt, ion, total carbon, total nitrogen, and total phosphorus contents and their ecological stoichiometric characteristics were measured and analyzed after theimplementation of different microtopographic modification patterns, with bare mudflats as the control.

**Results:**

The results showed that microtopographic modification significantly changed the soil water and salt contents and the soil total carbon, total nitrogen and total phosphorus contents. Compared with the bare ground, microtopographic transformation significantly promoted the growth of the pioneer plant *Suaeda salsa*, significantly increased the soil water and nutrient contents, and significantly decreased the soil salinity. The soil salinity was mainly reduced by Na^+^ and Cl^-^ ions. The soil salinity and nutrient contents gradually decreased with increasing soil depth, indicating the occurrence of surface aggregation. Compared to that of the bare ground, the soil C/N was significantly lower and the N/P was significantly higher in the microtopographic treatments, and the overall performance suggested soil N limitation. The ions contained in the saline soil were dominated by Na^+^ and Cl^-^, followed by Mg^2+^ and SO_4_^2-^, with lower contents of K^+^, Ca^2+^ and HCO_3_^-^. Among the six microtopography modification patterns, the crescent-shaped pattern best promoted vegetation restoration. This pattern was the most effective in reducing soil salinity, with a 98.53% reduction in soil salinity compared with that of bare ground, followed by the pin-shaped pattern. Compared with that in the bare ground samples, the nutrient content in the samples from the step-shaped modification increased by 23.27%; finally, the S-shaped, step-shaped and dense stripe-shaped patterns performed poorly in terms of plant restoration and soil improvement.

**Discussion:**

It is suggested that a crescent-shaped pattern should be considered first when carrying out microtopographic transformation on the beaches of the Yellow River Delta, followed by stripe-shaped and pin-shaped patterns. The dense strip-shaped should not be adopted.

## Introduction

Coastal mudflats in marine environments are formed by the combined effects of sedimentation, seawater scouring and tides (Finlayson, 1999). As a transitional area linking the land and sea, coastal mudflats are a key component of the natural ecosystem in the muddy coastal zone and are characterized by soil salinization, low plant cover, simple ecosystem structure and poor stability (Bruland and Richardson, 2005). The coastal mudflat transition zone has high carbon sequestration and storage capacities and large ability to protect tidal processes; these functions can purify water and improve the stability of biological species (Moxham et al., 2019; Murray et al., 2019; Xie et al., 2020) and are therefore of great importance in maintaining the balance of regional ecosystems (Cui et al., 2016).

The Yellow River Delta is a representative saline area in China and is located at the mouth of the Yellow River. The Yellow River Delta comprises 50.9% of the total saline soil area, and soil salinity in this region is quite severe (Yao and Yang, 2007). The coastal beach mudflats of the Yellow River Delta are mainly distributed in muddy coastal zones with silty substrate. These areas are affected by tides and evaporation and bare land with a dry surface and flat terrain forms easily; ground vegetation is sparse, and coastal mudflats are ecologically fragile areas with representative vegetation degradation (Bruland and Richardson, 2005). Soil salinization severely restricts plant growth, and low soil nutrient content limits plant nutrient uptake and utilization. Soil salinization has led to soil degradation and reduced productivity in the Yellow River Delta, seriously hindering the sustainable development of local land and ecological protection (Li et al., 2014). In recent years, secondary soil salinization has received widespread attention as a worldwide problem affecting resources and an ecological issue (Boulbaba et al., 2012). Green improvement and the comprehensive utilization of saline land play important roles in improving the ecological environment and promoting sustainable economic and social development (Long et al., 2016).

Studies have shown that soil salinization in the Yellow River Delta is mainly influenced by the groundwater level, mineralization and plant cover (Fan et al., 2010). The current methods used to enhance the quality of saline soils include physical, biological, chemical and integrated management measures (Xia et al., 2019). Among these management approaches, physical measures are a focal point of research. Physical improvement measures have rapid and significant results, are relatively safe and easy to promote and have other advantages (Hu et al., 2022). Physical measures are improvements made by adjusting the soil structure, such as deep plowing and solarization, microzone soil modification, raising the ground, straw mulching, and adding physical amendments (Yang et al., 2018). Microtopography is an important physical improvement measure used to improve soil quality, mainly by forming combinations of mounds and pits of different shapes and controlling the physical and chemical properties of the soil. This approach has an important impact on soil hydrology and plant restoration processes under different topographic conditions (Chen et al., 2007; Ilan et al., 2007).

Microtopographic transformation mainly focuses on the lack of actual natural conditions and involves purposely transforming and improving the original structure of the ground, forming microtopographic environments with different sizes and shapes, effectively increasing surface heterogeneity, and changing material transformation processes and transport pathways (Kamphorst et al., 2000; Appels et al., 2011; Wolf et al., 2011). The increase in the area of suitable habitats and changes in spatial patterns provide a variety of ecological niches for organisms, thus contributing to increases in species diversity (Yang et al., 2017; Liu et al., 2020). In recent years, scholars in China and elsewhere have explored the effects of microtopographic modifications on surface ecological processes, including artificial modification of substrata, valley development and silt dam construction in gully channels (Mayor et al., 2008). It was found that microtopographies such as septic terraces, large terrace fields and fish scale pits had different degrees of water storage and fertilizer retention (Cao et al., 2005; Hammad et al., 2006). The effect of interslope furrowed terraces on improving soil water content was significant (Ilan et al., 2007). Measures such as terracing, horizontal steps and fish scale pit preparation caused different degrees of changes in surface microelevation, roughness and slope. These modifications created microtopography and water catchment areas that served to impound surface runoff and increased rainfall infiltration, thereby curbing regional soil erosion (Mayor et al., 2008). Microtopographic modifications can alter surface microhabitats and local climatic conditions, affecting the structure and function of biological communities (Masatoshi et al., 1996).

The soil salinization in the muddy coastal zone of the Yellow River Delta is severe, and most areas are bare land without plant cover. The terrain is flat, and seeds do not easily settle when the wind speed is too high; as a result, it is difficult for plants to become established on the bare land, which enhances the challenges associated with ecological restoration. Previous studies in which microtopographic transformation models were applied mostly involve green space construction and landscape transformation and are dominated by erosion control studies. Fewer studies have been conducted on the ability of microtopography to suppress secondary soil salinization, promote plant growth and improve soils in coastal areas with high salinity in muddy coastal zones. In the coastal mudflat area of the Yellow River Delta, the lack of vegetation restoration through microtopographic transformation has led to poor interception of irrigation water and grass, low vegetation cover and nonsignificant effects of salt reduction and soil modification. It is not clear which microtopographic modification is optimal for promoting plant growth and soil improvement in muddy coastal zone bare mudflats.

The microtopographic modification method, in which the surface roughness is altered, was adopted, and six microtopographic modifications were applied. Based on the change pattern of vegetation growth, soil water and salt and nutrient characteristics after microtopographic modification, a model of suitable microtopographic modifications for saline land transformation in the region was developed. This research serves as a reference for vegetation and soil ecological restoration in coastal mudflat transition zones. A comprehensive evaluation of the microtopographic models suitable for coastal mudflats was conducted to provide technical support for vegetation restoration and ecological rehabilitation of coastal mudflats in the Yellow River Delta.

## Materials and methods

### Overview of the study area

**Figure 1:**
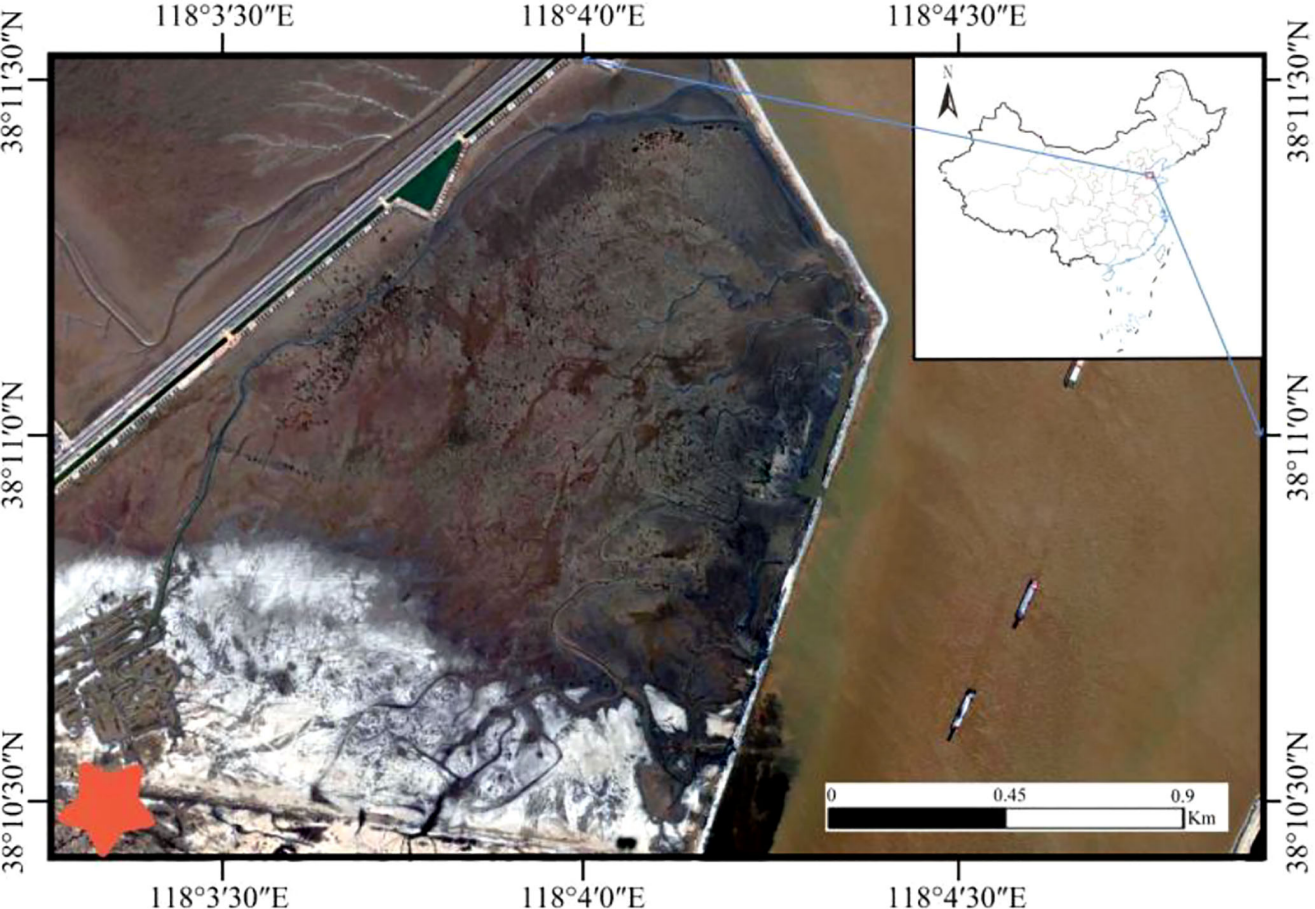
Study area in the Yellow River Delta.

The study area is located in the Natural *Tamarix ramosissima* forest in Binzhou Harbor (38°10′25-35″N, 118°03′05-15″E), Beihai Economic and Technological Development Zone, Binzhou city, Shandong Province, Yellow River Delta. The study area has a warm temperate continental monsoon climate (**Figure 1**), with four distinct seasons and uneven spatial and temporal distributions of precipitation. The annual average temperature is 12.2°C, and the annual average rainfall is 577.7 mm. Approximately 70% of the precipitation is concentrated in July and August, and the annual average evaporation is 1,806 mm, 51.7% of which occurs in spring. The groundwater table is shallow (1.2~2.4 m), and the average salinity reaches 32.4 g·L^-1^. In the study area, the terrain is low, flat and broad. The beach soil is mainly composed of marine leaching parent material at the bottom, and alluvial loess parent material is deposited on the top. The soil in the beach area is an alluvial loess matrix, mainly chalky sand and silty chalky sand; the soil is sandy and sticky and easy to compact, has poor ventilation and permeability by seawater, and a high content of soluble salts. The coastal mudflat area is affected by groundwater with high levels of mineralization and seawater intrusion, and the soil salt content can reach more than 30 g·kg^-1^ (Fan et al., 2010). Due to the shallow groundwater level, high salinity, and seawater intrusion, the soil soluble salt content in this area is high, and the extent of soil salinization is severe. The main plant species include T*amarix chinensis*, *Phragmites communis*, *Suaeda salsa*, *Aeluropus littoralis*, *Cynanchum chinense*, *Imperata cylindrica* and other salt-tolerant plants.

### Experimental design

**Figure 2:**
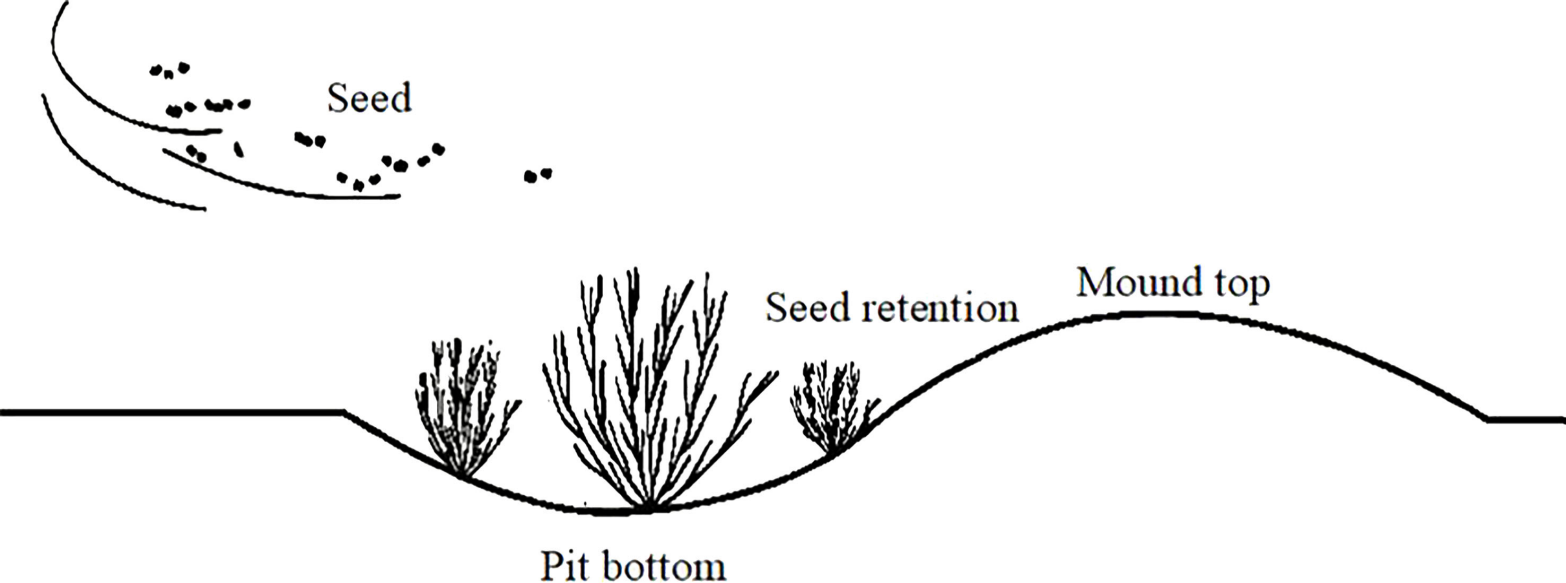
Source of inspiration for microtopographic modifications.

**Figure 3:**
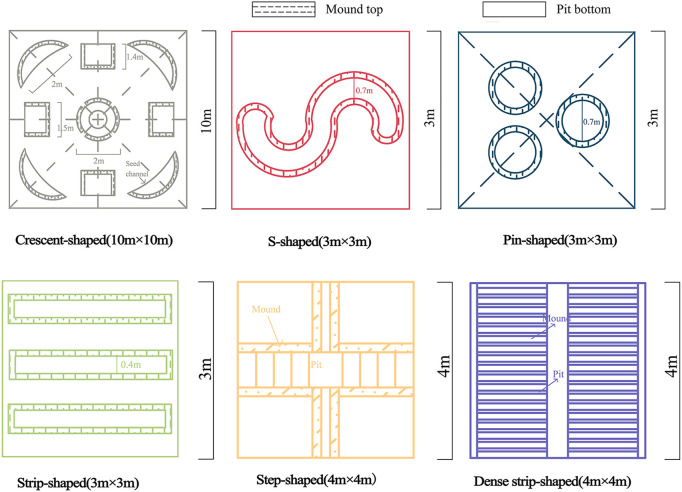
Schematic diagram of the microtopographic modifications.

In October 2018, six microtopographic transformation models with different specifications were implemented on the bare beach of the test site, and the bare beach (CK) was used as the control. Three replicates were established for each microtopographic transformation model, and the specific microtopographies were as follows: in the pin-shaped microtopography, three circular mounds with pits with a diameter of 0.7 m and a depth of 0.25 m were established in a 3 m×3 m quadrat; in the S-shaped microtopography, an S-shaped mound pit assembly with a width of 0.4 m and a depth of 0.2 m was established in a 3 m×3 m quadrat; in the strip-shaped microtopography, three strip-shaped mound pits with a length of 3 m, a width of 0.4 m and a depth of 0.3 m were established on a 3 m×3 m spline; in the step-shaped microtopography, different depths were established on 4 m×4 m quadrats to form stepped mounds and pits with depths of 0.1 m, 0.2 m and 0.3 m; in the dense strip-shaped microtopography, two dense strip mound pits with a width of 1.6 m, a length of 4 m and a depth of 0.05 m were established in a 4 m×4 m quadrat; and in the crescent-shaped microtopography, in a 10 m×10 m quadrangle, there were 9 crescent shapes, rectangles and round pits with different orientations and depths of 0.5 m. See **Figure 2** and **Figure 3** for details on the specific design shapes and parameters. Different specifications and shapes of the microtopographies were created mainly with a spade. The artificially excavated soil was cultivated into mounds, and the resulting hole was left to provide a location for seed interception. All mounds were of the same height, and the mounds intercepted seeds floating in the air, causing the seeds to then land in the pit. The soil pit was set up to store plant seeds, and a 10 cm thick layer of soil was loosened at the bottom of the pit to facilitate plant root germination and improve the germination rate of seeds.

After a two-year restoration process, the plant species, biomass, coverage, density and other indicators of the six microtopographic reconstruction models were determined in October 2020. Soil samples from the 0-10 cm and 10-20 cm soil layers were collected from the bottom of pits in plant-growing areas with different microtopographic transformation models. Three soil samples were randomly collected from each soil layer and mixed to form a composite sample, and three replicate soil samples were collected from each microtopographic transformation model.

### Sample collection and determination

The plant cover survey method used was the point sampling method, the density survey method used was the equidistant sampling method, and the biomass was measured by drying and weighing the samples. The collected soil was transported to the laboratory, dried and screened, and the soil indicators were tested. The soil water content was determined by the drying method. Soil salinity was determined by the residue drying method. Soil pH was determined by a pH meter. Total phosphorus (TP) was determined by HClO_4_-H_2_SO_4_ digestion and the molybdenum blue colorimetric method. Total carbon (TC) was determined by the K_2_Cr_2_O_7_-H_2_SO_4_ oxidation method. Total nitrogen (TN) was determined by the Kjeldahl method. The soil potassium ion (K^+^) and sodium ion (Na^+^) contents were determined by the flame photometric method. The calcium ion (Ca^2+^) and magnesium ion (Mg^2+^) contents were determined by the EDTA titration method. The bicarbonate ion (HCO_3_^-^) content was determined by the double indicator neutralization titration method. The chlorine ion (Cl^-^) content was determined by the silver nitrate titration method. The sulfate ion (SO_4_^2-^) content was determined by the EDTA indirect complexation titration method.

### Data analysis and processing

AutoCAD 2016 was used to draw the schematic diagram of the sample plot. Excel 2010 was used to process the experimental data. One-way analysis of variance (one-way ANOVA) and multiple comparisons (Duncan’s test) were performed on soil physicochemical indicators using SPSS 26.0 statistical software (*P* < 0.05). T tests were performed on different soil layers with the same microtopography (α=0.05). The plots were created using Origin 2021. The data in the chart are the means ± standard errors.

## Results

### Growth characteristics of *Suaeda salsa*

In each of the different microtopographic modification patterns, only *Suaeda salsa* was present at the bottom of the pit. As shown in **Table 1**, The biomass of *S. salsa* was significantly higher after microtopography modification than in the CK (*P* < 0.05). The biomass of *S. salsa* was highest in the sample from the crescent-shaped microtopography, followed by the step-shaped microtopography, and lowest in the sample from the dense strip-shaped microtopography. The samples from the step-shaped, strip-shaped, pin-shaped, S-shaped and dense strip-shaped microtopographies had 19.86%, 24.16%, 44.58%, 51.61%, and 86.70% lower biomasses, respectively, than those from the crescent-shaped microtopography. The plant density was highest in the crescent-shaped microtopography. The plant densities of the strip-shaped, pin-shaped, step-shaped, S-shaped and dense strip-shaped microtopographies were 6.32%, 11.76%, 21.76%, 26.47%, and 85.29% lower, respectively, than those of the crescent-shaped microtopography. The plant coverage was highest in the crescent-shape microtopography. The plant coverage in the stripe-shaped, pin-shaped, step-shaped, S-shaped and dense strip-shaped microtopographies was 6.32%, 11.76%, 21.76%, 26.47%, and 85.29%, respectively, lower than that in the crescent-shaped microtopography.

**Table 1:** Growth characteristics of *Suaeda salsa* after different microtopographic modifications.

Microtopographic reconstruction mode	Plant biomass /g·m^-2^	Plant density/(10^2^·m^-2^)	Plant coverage /%
Strip	48.18 ± 2.60^b^	6.37 ± 0.85^a^	89.32 ± 0.41^a^
Pin	35.21 ± 1.53^b^	6.00 ± 0.73^a^	82.20 ± 0.25^a^
S	30.74 ± 2.60^b^	5.36 ± 0.54^b^	63.34 ± 1.04^b^
Dense	8.45 ± 0.01^c^	1.00 ± 1.21^c^	18.21 ± 0.28^c^
Step	50.91 ± 0.94^a^	5.00 ± 0.35^b^	70.31 ± 0.67^b^
Crescent	63.53 ± 0.94^a^	6.80 ± 1.01^a^	98.21 ± 1.42^a^

Different letters in the same column indicate significant differences between different microtopography types (P < 0.05).

### Soil water and salt contents

**Figure 4:**
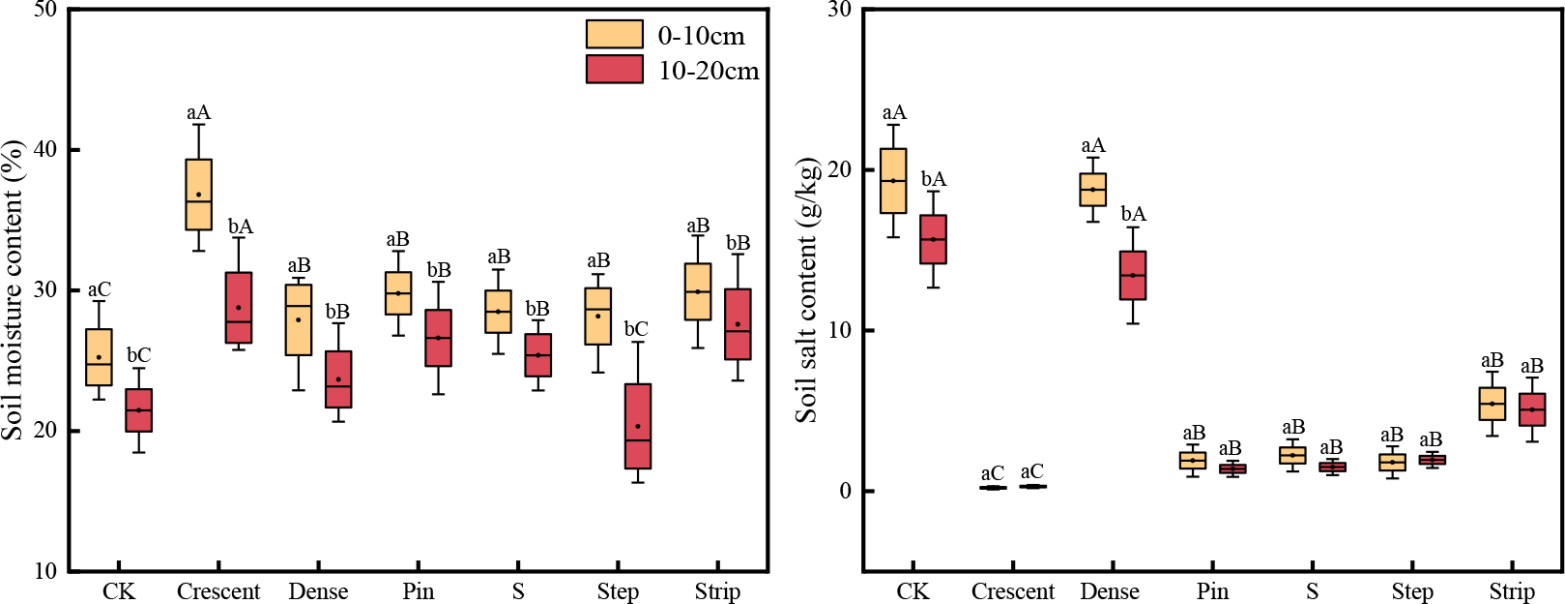
Soil water content and salt content in different microtopographic transformation models. Note: Different capital letters indicate significant differences between microtopographies, while difference lowercase letters indicate significant differences between soil layers (*P* < 0.05).

As shown in **Figure 4**, the soil water contents of samples from different microtopographic modifications were significantly higher in the 0-10 cm soil layer than in the 10-20 cm soil layer. However, the difference in water content between soil layers showed some variability with different microtopographic treatments. The soil water content in the 0-10 cm soil layer in the sample from the crescent-shaped was 27.99% higher than that in the 10-20 cm soil layer. The smallest difference was observed for the strip-shaped microtopography. The soil water content was significantly higher after microtopographic modification than in the CK (*P* < 0.05). The water content in the 0-20 cm soil layer showed the following order: crescent-shape > strip-shaped > pin-shaped > S-shaped > dense strip-shaped > CK > step-shaped. Compared to that in the CK, the soil water content increased by 34.02%, 28.52%, 24.00%, 15.94% and 10.25% in the five microtopographic modification patterns, respectively. The water content in the terraced soil was reduced by 5.26% compared to that in the CK.

Soil salinity was higher in the 0-10 cm soil layer than in the 10-20 cm soil layer for all the microtopographic treatments except the step-shaped and crescent-shaped microtopographies, in which the salinity levels were lower in the 0-10 cm soil layer than in the 10-20 cm soil layer. This result indicated that the soil salinity was strongly aggregated at the surface. The soil salinity was significantly lower after microtopography modification than in the CK (*P* < 0.05). The salinity in the 0-10 cm layer of the soils in the dense strip-shaped microtopography was 28.44% higher than that in the 10-20 cm layer, while the strip-shaped pattern showed the smallest difference in salinity between soil depths. The soil salinity in the 0-20 cm soil layer was as follows: crescent-shaped < pin-shaped < S-shaped < step-shaped < strip-shaped < dense strip-shaped < CK. Compared with that in the CK, the soil salinity was reduced by 14.29%, 67.60%, 87.57%, 90.41%, 91.13% and 98.14% in the six microtopography modification patterns.

**Figure 5:**
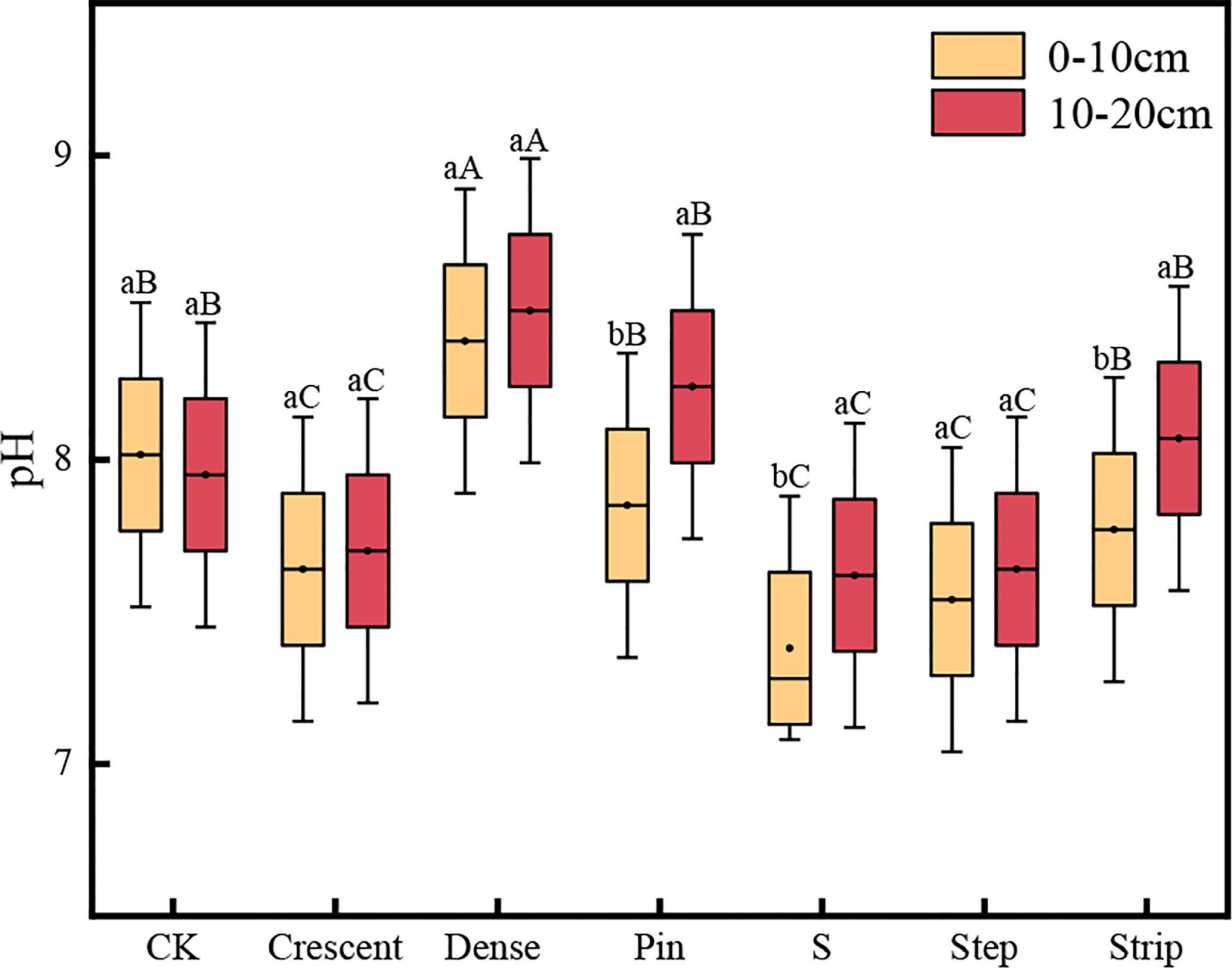
Soil pH in the microtopographic transformation models. Note: Different capital letters indicate significant differences between microtopographies, while different lowercase letters indicate significant differences between soil layers (*P* < 0.05).

As shown in **Figure 5**, the soil pH was higher in the 0-10 cm soil layer than in the 10-20 cm soil layer for all the microtopographies. However, the pH showed some variability between soil layers among the different microtopographic treatments. The differences in pH between soil layers were significantly greater among the strip-shaped, S-shaped and pin-shaped microtopographies than in the CK (*P* < 0.05). Soil pH was significantly lower in the step-shaped, S-shaped and crescent-shaped microtopographies than in the CK (*P* < 0.05). The ranking of soil pH in the 0-20 cm was as follows: crescent-shaped < pin-shaped < S-shaped < step-shaped < strip-shaped < dense strip-shaped < CK.

### Major cation content in the soil

**Figure 6:**
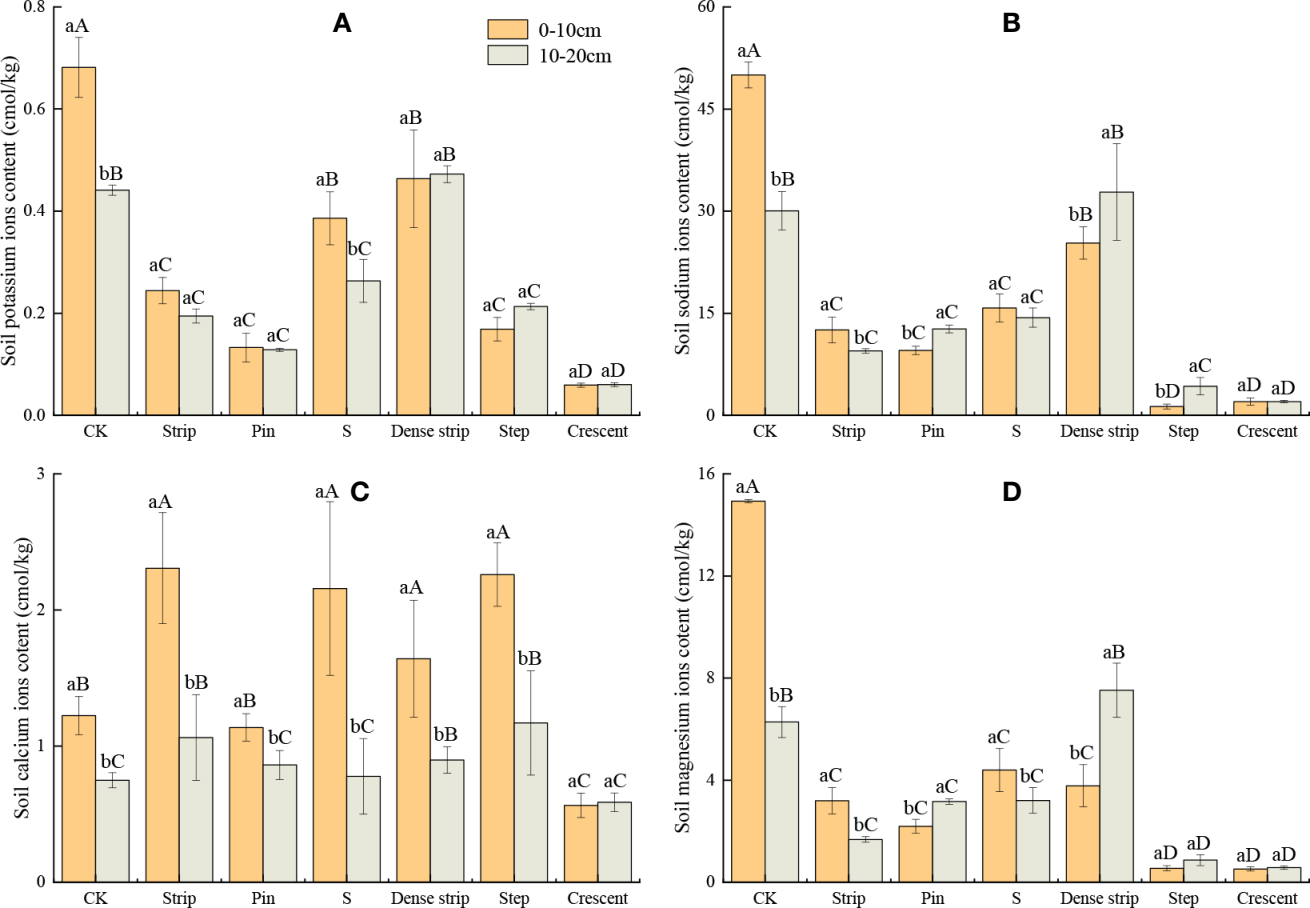
Soil salt potassium ions content **(A)**, soil salt sodium ions content **(B)**, soil salt calcium ions content **(C)**, and soil salt magnesium ions content **(D)** in different microtopographic modification models. Note: Different capital letters indicate significant differences between microtopographies, while different lowercase letters indicate significant differences between soil layers (*P* < 0.05).

As shown in **Figure 6**, the soil cation content after the different microtopographic treatments differed significantly with the soil layer. There was no significant difference in the K^+^ content among the soil layers for any of the microtopographies (*P* < 0.05) except the S-shaped microtopography, in which the K^+^ content was significantly higher in the 0-10 cm soil layer than in the 10-20 cm soil layer (*P* < 0.05). The Na^+^ content in the 0-10 cm soil layer was significantly lower than that in the 10-20 cm soil layer in the pin-shaped, step-shaped, and dense strip-shaped microtopographies (*P* < 0.05). The Na^+^ content in the strip-shaped microtopography, however, was significantly higher in the 0-10 cm soil layer than in the 10-20 cm soil layer (*P* < 0.05). The soil Ca^2+^ content was significantly higher in the 0-10 cm soil layer than in the 10-20 cm soil layer in all the microtopographic treatments except that with a crescent-shaped microtopography (*P* < 0.05). In the soils from the pin-shaped and dense strip-shaped microtopographies, the Mg^2+^ content in the 0-10 cm soil layer was significantly lower than that in the 10-20 cm layer (*P* < 0.05). However, in the strip-shaped and S-shaped treatments, the soil Mg^2+^ contents were significantly higher in the 0-10 cm soil layer than in the 10-20 cm soil layer (*P* < 0.05).

The K^+^, Na^+^, and Mg^2+^ contents in the 10-20 cm soil layer were significantly lower after the microtopographic treatments were applied than in the CK (*P* < 0.05). The Ca^2+^ content after microtopographic modification was significantly higher in the 10-20 cm soil layer than in the CK, except in the crescent-shaped treatment (*P* < 0.05). The soil K^+^, Na^+^, and Mg^2+^ contents were significantly lower after microtopographic modification than in the CK (*P* < 0.05). The soil Ca^2+^ contents were higher in all the microtopographic treatments except for the crescent-shaped treatment than in the CK (*P* < 0.05). Compared with the bare ground, the K^+^, Mg^2+^, and Ca^2+^ contents in the 0-10 cm soil layer were lowest in the crescent-shaped microtopography, with values that were 91.31%, 96.53%, and 53.88% lower, respectively. The soil Na^+^ content was lowest in the step-shaped treatment, with a 97.41% reduction compared to that in the CK.

The ranking of K^+^ content in the 0-20 cm soil layer was as follows: crescent-shaped < pin-shaped < strip-shaped < step-shaped < S-shaped < dense strip-shaped < CK. The ranking of Na^+^ content in the 0-20 cm soil layer was as follows: crescent-shaped < step-shaped < strip-shaped < pin-shaped < CK < S-shaped < dense strip-shaped. The ranking of Ca^2+^ content of the 0-20 cm soil layer was as follows: crescent-shaped < S-shaped < pin-shaped < dense strip-shaped < strip-shaped < step-shaped < CK. The ranking of Mg^2+^ content of the 0-20 cm soil layer was as follows: crescent-shaped < step-shaped < strip-shaped < pin-shaped < S-shaped < dense strip-shaped < CK.

### Major anion content in the soil

**Figure 7:**
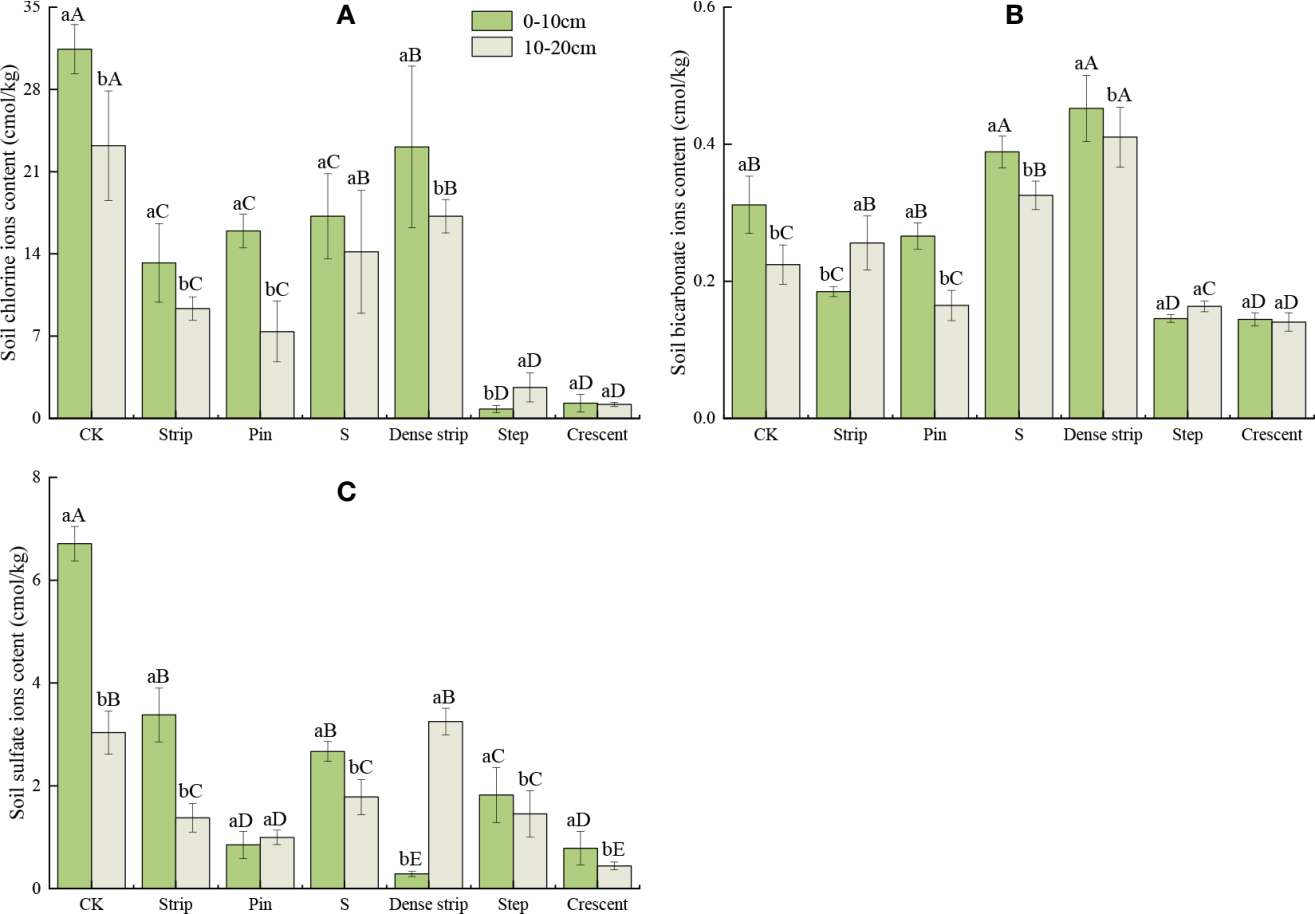
Soil chlorine ions contents **(A)**, soil bicarbonate ions contents **(B)** and soil sulfate ions contents **(C)** in different microtopographic modification models. Note: Different capital letters indicate significant differences between microtopographies, while different lowercase letters indicate significant differences between soil layers (*P* < 0.05).

As shown in **Figure 7**, the soil anion content after microtopographic treatment differed significantly by soil layer. The Cl^-^ content in the step-shaped microtopography was significantly higher in the 10-20 cm than in the 0-10 cm soil layer (*P* < 0.05), while there was no significant difference between soil layers for the crescent-shaped microtopography (*P* < 0.05). In the soils of the remaining four microtopographic treatments, the Cl^-^ contents were significantly lower in the 10-20 cm soil layer than in the 0-10 cm soil layer (*P* < 0.05). There was no significant difference in the HCO_3_^-^ contents in the 10-20 cm soil layer of the crescent-shaped and step-shaped microtopographies (*P* < 0.05). The soils in the strip-shaped microtopography showed a significantly higher HCO_3_^-^ content in the 0-10 cm soil layer than in the 10-20 cm soil layer (*P* < 0.05). The soil HCO_3_^-^ content in the remaining three microtopographic treatments was significantly lower in the 10-20 cm soil layer than in the 0-10 cm soil layer (*P* < 0.05). In the pin-shaped microtopographic treatment, there was no significant difference in the SO_4_^2-^ content between the soil layers (*P* < 0.05). In the dense strip-shaped treatment, the SO_4_^2-^ content was significantly lower in the 0-10 cm soil layer than in the 10-20 cm soil layer (*P* < 0.05). The SO_4_^2^- contents of the remaining four microtopographic treatments were significantly lower in the 10-20 cm soil layer than in the 0-10 cm soil layer (*P* < 0.05).

The soil Cl^-^ and SO_4_^2-^ contents were significantly lower after microtopographic modification than in the CK (*P* < 0.05). The HCO_3_^-^ contents in the S-shaped and dense strip-shaped treatments were significantly higher than that in the CK (*P* < 0.05); however, in the crescent-shaped and step-shaped treatments, the HCO_3_^-^ contents were significantly lower than that in the CK (*P* < 0.05). Compared with the CK, the Cl^-^, HCO_3_^-^, and SO_4_^2-^ contents in the 0-10 cm soil layer were lowest in the step-shaped, crescent-shaped, and dense strip-shaped treatments, with reductions of 97.44%, 53.66%, and 95.73%, respectively.

The ranking of Cl^-^ contents in the 0-20 cm soil layer was as follows: crescent-shaped < step-shaped < pin-shaped < strip-shaped < S-shaped < dense strip-shaped < CK. The ranking of HCO_3_^-^ contents in the 0-20 cm soil was as follows: crescent-shaped < step-shaped < pin-shaped < strip-shaped < CK < S-shaped < dense strip-shaped. The ranking of SO_4_^2-^ contents in the 0-20 cm soil was as follows: crescent-shaped < pin-shaped < step-shaped < strip-shaped < S-shaped < dense strip-shaped < CK.

### Soil nutrients

**Figure 8:**
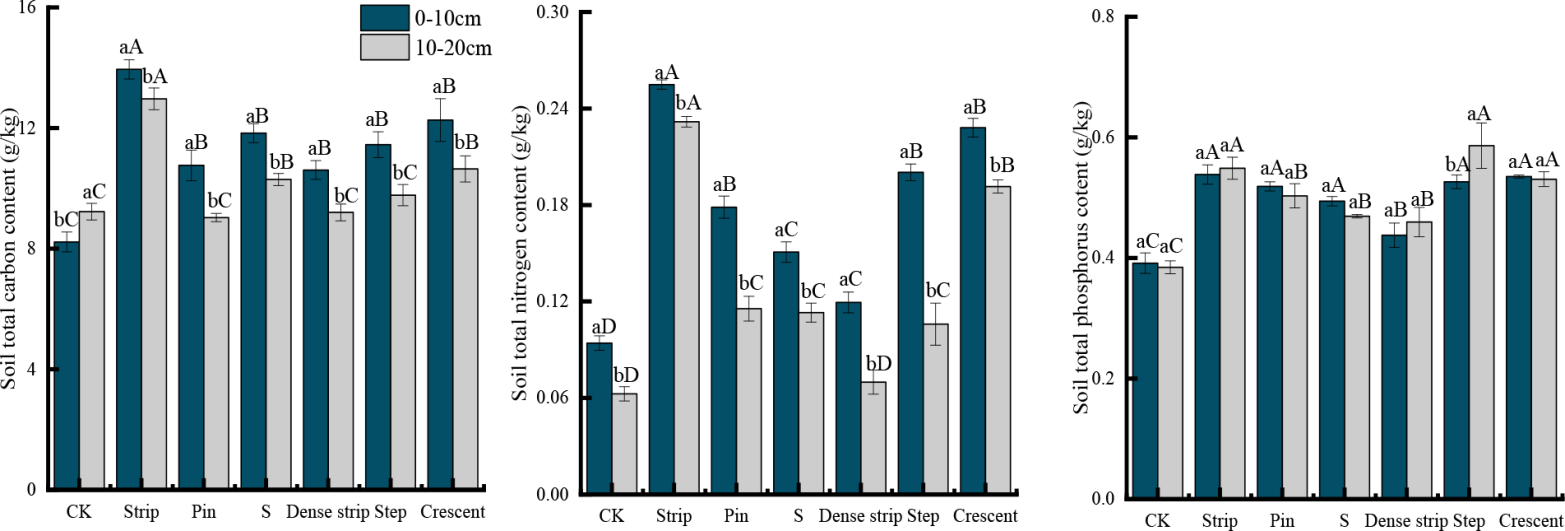
Soil total carbon, total nitrogen, and total phosphorus contents in the different microtopographic transformation models. Different capital letters indicate significant differences between microtopographies, while different lowercase letters indicate significant differences between soil layers (*P* < 0.05).

As shown in **Figure 8**, the soil TC and TN contents in the different microtopographic modifications were significantly higher in the 0-10 cm soil layer than in the 10-20 cm soil layer (*P* < 0.05). The differences in the soil TP contents between the five microtopographic treatments and the CK were not significant (*P* < 0.05), except for the step-shaped treatment. There was no significant change in the differences in the TC and TP contents between the 0-10 cm and 10-20 cm soil layers of the six microtopographic treatments compared to that in the CK (*P* < 0.05). However, the TN contents in the 0-10 cm and 10-20 cm soil layers showed some variability among the microtopographic treatments. The difference in the TN contents between the 0-10 cm and 10-20 cm soil layers was significantly higher for the pin-shaped, dense strip-shaped, and step-shaped treatments than for the other treatments (*P* < 0.05). The largest difference was observed in the step-shaped treatment. The TN content in the 0-10 cm soil layer was 47.13% higher than that in the 10-20 cm soil layer. There was no significant change in the magnitude of the difference in the TN contents for the remaining three microtopographic treatments between the 0-10 cm and 10-20 cm soil layers (*P* < 0.05).

The TC, TN, and TP contents in the soil were significantly higher after microtopographic modification than in the CK (*P* < 0.05). The soil TC and TN contents in the different microtopographies were highest in the strip-shaped treatment, with values 35.17% and 211.92% higher, respectively, than those in the CK. The soil TP content was highest in the step-shaped treatment, with an increase of 45.10% compared to that in the CK.

The ranking of TN contents in the 0-20 cm soil layer was as follows: strip-shaped > crescent-shaped > S-shaped > step-shaped > dense strip-shaped > pin-shaped > CK. The ranking of TC contents in the 0-20 cm soil layer was as follows: strip-shaped > crescent-shaped > step-shaped > pin-shaped > S-shaped > dense strip-shaped > CK. The ranking of TP contents in the 0-20 cm soil was as follows: step-shaped > strip-shaped > crescent-shaped > pin-shaped > S-shaped > dense strip-shaped > CK.

### Soil CNP stoichiometry

As shown in **Table 2**, the soil ecological stoichiometry characteristics after different microtopography treatments differed significantly with soil layer. The soil C/N in the 0-10 cm layer was lower than that in the 10-20 cm layer and significantly lower than that in the CK (*P* < 0.05). The C/P and N/P in the 0-10 cm soil layer were higher than those in the 10-20 cm soil layer. The ranges of variation in the soil C/N, C/P, and N/P were 53.70-149.82, 16.80-25.96, and 0.15-0.47, respectively, for the six microtopographic modification models. Except for the strip-shaped and dense strip-shaped treatments, the C/P in the soils with microtopographic treatments was significantly lower than that in the CK (*P* < 0.05). The soil N/P after microtopographic modification was significantly higher than that in the CK (*P* < 0.05). The soil C/N was lowest in the crescent-shaped treatment, and C/P was lowest in the step-shaped treatment, with values that were 53.97% and 14.56% lower than that in the CK, respectively. Soil C/P and N/P were both highest in the strip-shaped soil, with values that were 9.97% and 122.50% higher than that in the CK, respectively.

**Table 2:** Soil ecological stoichiometry in different microtopographic alteration models.

Microtopography modification model	Soil depth	Soil C/N	Soil C/P	Soil N/P
CK	0-10 cm	87.48 ± 2.91^bA^	21.06 ± 0.96^aB^	0.24 ± 0.01^aC^
	10-20 cm	149.82 ± 16.12^aA^	24.06 ± 1.35^aA^	0.16 ± 0.01^bD^
Strip	0-10 cm	54.69 ± 0.66^aD^	25.96 ± 1.23^aA^	0.47 ± 0.19^aA^
	10-20 cm	55.93 ± 1.06^aD^	23.66 ± 0.83^bA^	0.42 ± 0.02^aA^
Pin	0-10 cm	60.34 ± 2.77^bC^	20.73 ± 0.79^aB^	0.34 ± 0.02^aB^
	10-20 cm	79.07 ± 6.70^aC^	18.03 ± 1.95^bB^	0.23 ± 0.01^bC^
S	0-10 cm	78.62 ± 1.78^bB^	23.94 ± 0.48^aB^	0.31 ± 0.01^aB^
	10-20 cm	91.65 ± 3.35^aB^	21.95 ± 0.48^aA^	0.24 ± 0.01^bC^
Dense strip	0-10 cm	89.11 ± 3.10^bA^	24.26 ± 0.47^aB^	0.27 ± 0.01^aC^
	10-20 cm	134.28 ± 12.25^aA^	20.13 ± 1.19^aA^	0.15 ± 0.02^bD^
Step	0-10 cm	57.12 ± 0.76^bD^	21.75 ± 0.67^aB^	0.38 ± 0.01^aB^
	10-20 cm	94.45 ± 8.93^aB^	16.80 ± 1.22^bB^	0.18 ± 0.01^bD^
Crescent	0-10 cm	53.70 ± 2.05^aD^	22.92 ± 1.27^aB^	0.43 ± 0.02^aA^
	10-20 cm	55.54 ± 1.56^aD^	20.04 ± 0.51^aA^	0.36 ± 0.01^bB^

Different capital letters indicate significant differences between microtopographies, while different lowercase letters indicate significant differences between soil layers (P < 0.05).

The ranking of C/N in the 0-20 cm soil layer was as follows: crescent-shaped < strip-shaped < pin-shaped < step-shaped < S-shaped < dense strip-shaped < CK. The ranking of C/P in the 0-20 cm soil layer was as follows: strip-shaped > S-shaped > CK > dense strip-shaped > crescent-shaped > pin-shaped > step-shaped. The N/P in the 0-20 cm layer soil was as follows: strip-shaped > crescent-shaped > pin-shaped > step-shaped > S-shaped > dense strip-shaped > CK.

### Evaluation of the combined effect of different microtopography modification models

**Figure 9:**
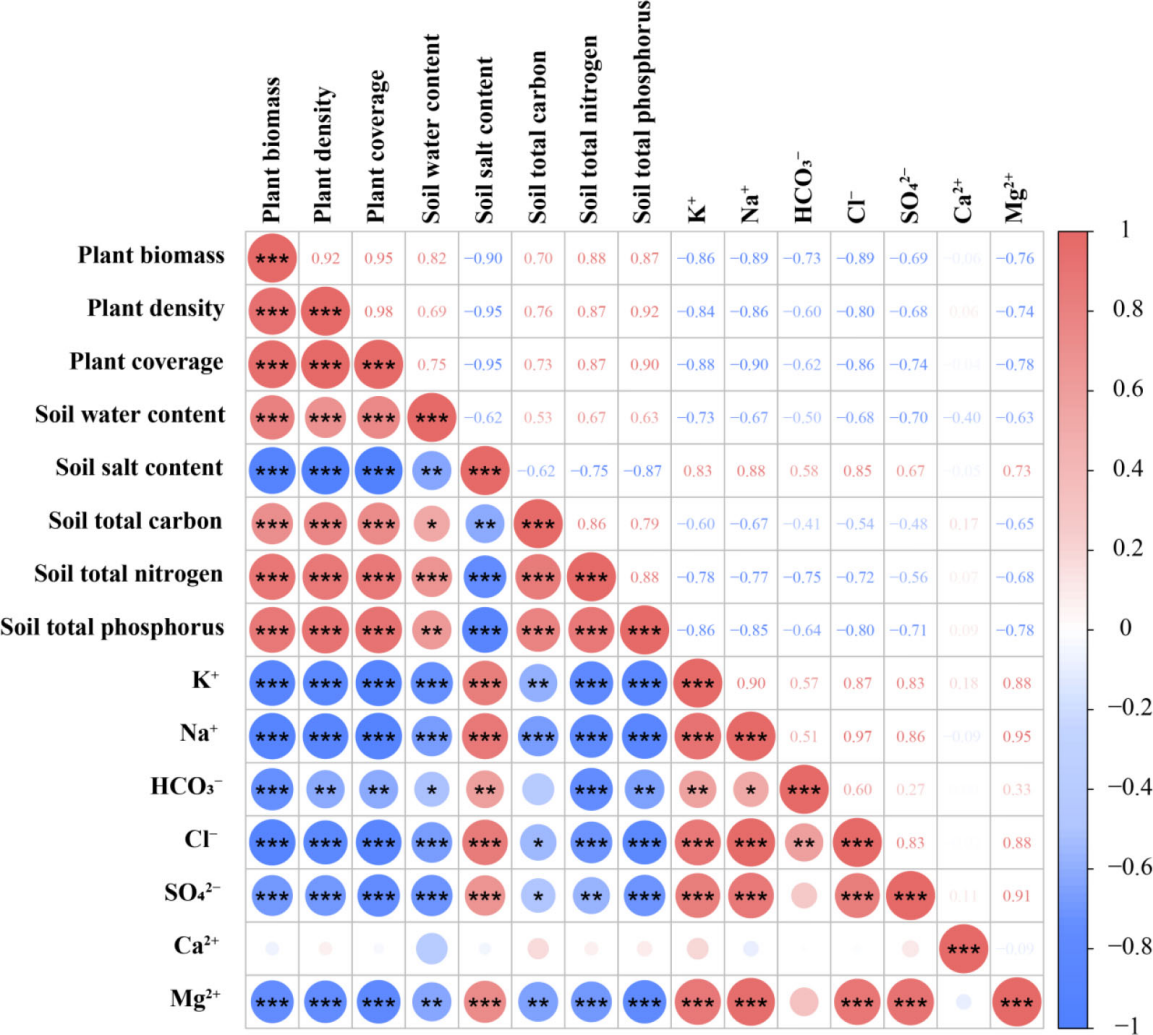
Correlation coefficients of soil water and salt characteristics, soil chemical properties and soil salt ions under different microtopographic transformation models. *, **, *** indicate that the correlation is significant at 0.05, 0.01 and 0.001 level, respectively.

As shown in **Figure 9**, plant growth indicators were significantly positively and negatively correlated with soil water content and salinity, respectively. Soil water content was highly significantly and positively correlated with total carbon, total nitrogen, and total phosphorus and highly and negatively correlated with soil salinity and K^+^, Na^+^, HCO^3-^, Cl^-^, SO_4_^2-^, and Mg^2+^ contents. Soil salinity had a highly significant positive correlation with the contents of K^+^, Na^+^, HCO^3-^, Cl^-^, SO_4_^2-^, and Mg^2+^ and a highly significant negative correlation with total carbon, total nitrogen, and total phosphorus. Except for Ca^2+^, all the major ions (K^+^, Na^+^, HCO^3-^, Cl^-^, SO_4_^2-^, and Mg^2+^) were all significantly and positively correlated with soil salinity.

## Discussion

### Effects of microtopographic modification on plants, soil water contents and soil salinity

Soil water and salt composition is an important indicator of coastal soil salinization and a necessary reference indicator for the improvement of coastal salinized soil (Cui et al., 2022). Topographic factors have an important influence on soil water content (Yang et al., 2015). Microtopographic modifications altered the moisture conditions of the bare ground in the muddy coastal zone, resulting in a significant increase in soil moisture content. The warm zone formed at the bottom of the pit after the microtopographic modification in this study allowed the rapid establishment and growth of *S. salsa*. The plant coverage in the crescent-shaped soil treatment reached 98.21%, which was significantly improved compared with that in bare ground area of the beach (*P* < 0.05). Increased plant coverage can reduce the evaporation of soil water (Liancourt et al., 2005; Cui et al., 2018). The biomass of *S. salsa* was highly significantly and positively correlated with plant density and coverage and soil water content (*P* < 0.001). The biomass of *S. salsa* in saline areas was highly significantly and negatively correlated with soil salinity (*P* < 0.001). The density of *S. salsa* in saline areas was highly significantly and positively correlated with coverage and soil water content (*P* < 0.001) and highly significantly and negatively correlated with soil salinity (*P* < 0.001). The *S. salsa* cover was highly significantly and positively correlated with the soil water content (*P* < 0.001) and highly significantly and negatively correlated with soil salinity (*P* < 0.001). Improvements in microenvironmental conditions such as soil water content and soil salinity under the six microtopographic modification patterns promoted the plant density and biomass of *S. salsa*.

Low-lying terrain created by microtopography is more prone to resource accumulation. The fertilizer island effect within circular patches acts as a positive plant−soil feedback (An et al., 2008). This process results in a nonuniform spatial distribution of soil water content, but the differences among treatments were not significant. This result may be related to the shallow depth of the soil layer in the experimental area. The optimal soil salt concentration for *S. salsa* growth is 12.14 g·kg^-1^, and the optimal water content is 59.82% (Wang et al., 2014). *S. salsa* grew vigorously in the bottom of the pits with high salinity. The stem and leaf mulch of this species inhibit soil desalination (Flowers and Colmer, 2010). *S. salsa* absorbs large amounts of salt ions through its root system during growth (Zhao et al., 2003), thus reducing the salt content in the soil in the pit bottom. The crescent-shaped pattern showed the most significant performance in reducing soil salinity at the bottom of the pit, with a 98.59% reduction compared to that in soil from the bare ground at the beach. Related studies also found that microtopography could improve rainfall infiltration and water harvesting and storage (Bergkamp, 1998; Appels et al., 2011; Courtwright and Findlay, 2011). Microtopography mainly changes the surface water infiltration capacity and retention time by changing the surface relief and diffuse flow connectivity (Chen et al., 2007; Yang et al., 2015). These changes can significantly increase the soil water content under certain conditions. Microtopography is prone to surface elevation changes. Microtopography changes the surface roughness by increasing the surface area. The six microtopographic modification patterns increased the roughness of the flat bare ground of the coastal mudflats in the muddy coastal zone. The enrichment of rainwater at the bottom of the pit increased the surface water storage, and this change caused a significant increase in the water content of the soil in the pit bottom. The crescent-shaped pattern showed the most significant performance in elevating the soil water content at the bottom of the pit, with an increase of 40.41% compared to that in the bare ground on the beach. Soil salinity had a highly significant negative correlation with water content. The soil salinity showed a significant change after microtopographic modification, and the soil salinity decreased with increasing soil layer.

### Effects of microtopographic modification on soil ion exchange

The dissolved salt ion concentration provides a comprehensive picture of the soil salt content. Most calcium salts are not easily soluble in water. With the exception of Ca^2+^, the contents of all the major ions (K^+^, Na^+^, Cl^-^, HCO_3_^-^, SO_4_^2-^, and Mg^2+^) were significantly and positively correlated with soil salinity. The percentages of soil Cl^-^ and SO_4_2^-^ and Na^+^ and Mg^2+^ cations varied with microtopography, accounting for 84.16%, 15.01%, 76.72%, and 20.33% of the anions and cations, respectively. The proportions were relatively similar. This result indicated that the salinity of the coastal mudflats in the Yellow River Delta is dominated by chlorides and sulfides. Moreover, evaporation pressure is strong in the Yellow River Delta, and salts in groundwater accumulate at the surface as the capillary water rises. The movement of salts in groundwater is greatest for chloride, followed by sulfate, while carbonate is most stable. The control of soil Na^+^ and Cl^-^ should be emphasized in the remediation of salinized coastal mudflat soils in muddy coastal zones. Soil salinity can be reduced with engineering and by supporting bioremediation measures.

The muddy coastal zone mudflats have a shallow water table and intense evaporation pressure. These conditions cause salt-based ions in the soil to move to the surface with the evaporation of groundwater, where they accumulate (Chu et al., 2017; Luna et al., 2019). The microtopographic surface relief allows the salt at the bottom of the pits to move to the top of the mounds as the groundwater evaporates. The pooling of rainwater at the bottom of the pits results in a higher water content and a lower soil salinity (Hammad et al., 2006). The salt contents, i.e., Na^+^ and Cl^-^, of the soils in different soil layers were significantly reduced after microtopography modification. This result may be due to the increased surface relief and faster groundwater flow due to microtopographic modifications, such that salts were less likely to accumulate (Hammad et al., 2006). Microtopography influences the variation in soil salinity by affecting surface hydrological connectivity, so soil salinity exhibits large differences in spatial distribution over a small regional scale (Qureshi et al., 2013). This condition results in the soil having a higher water content and a lower salinity. *S. salsa* grew vigorously in the study area, and the plant growth indicators were significantly positively and negatively correlated with soil water content and salinity, respectively.

### Effect of microtopographic modifications on soil nutrients

Carbon, nitrogen, and phosphorus are the main indicators of nutrients in soil. Some results have shown that microtopography affects water storage and fertilization (Liu et al., 2020). The six microtopographic modification models implemented in this study improved the soil nutrient conditions in the bare areas of the muddy coastal zone. The terrain was influenced by wind, which carries sand and deposits sand particles on the bottom of the pits. Nutrients were lost from the top of the mounds to the bottom of the pits, and this process increased the accumulation of nutrients at the bottom of the pits (Fernandez-Montblanc et al., 2020). The improvement in water and salt conditions facilitated the germination and growth of seeds at the bottom of the pits (Zhang et al., 2004), and the environmental conditions resulted in significant increases in TC, TN and TP in the soil. Of the six microtopography patterns, the strip-shaped pattern was the most effective in increasing soil nutrients. The TC and TN in the soils of the strip-shaped treatment increased by 35.17% and 211.92%, respectively, compared to those in the CK. The spatial distribution of soil nutrients in the microtopographic treatments was not uniform, and the distribution indicated surface aggregation. This was due to the germination and growth of *S. salsa* in the saline area after microtopographic transformation; the root system was buried at a shallow depth, and the accumulation of plant apoplast and root absorption occurred (Wu et al., 2018; Wang et al., 2021).

Han et al. (2011) found that growth indicators of 1900 Chinese plants were positively correlated with nutrient contents such as carbon, nitrogen, and phosphorus. Soil C/N was shown to be inversely proportional to the rate of organic matter decomposition (Liu et al., 2020). In this study, soil C/N was significantly lower after microtopographic modification and was significantly negatively correlated with soil nutrient contents. Soil C/P is a reflection of the phosphorus released from decomposing organic matter and the phosphorus sequestration potential of the soil (Liu et al., 2020). Soil N/P is an indicator of the abundance or deficiency of the soil nutrient supply (Liu et al., 2020). Soil N/P was significantly higher after microtopographic modification and was positively correlated with plant growth indicators. The increase in apoplastic fluid and humus at the bottom of the pits also contributed to the increase in soil N/P. Soil nitrogen is the main limiting factor affecting plant growth (Muneer et al., 2020). According to the results of Tessier and Raynal, N/P can be used as an indicator for the diagnosis of nitrogen saturation (Tessier and Raynal, 2003). The mean levels of soil C/N, C/P, and N/P in China were 61.0 ( ± 0.1), 11.9 ( ± 0.1), and 5.2 ( ± 0.1), respectively (Tian et al., 2010). These findings indicate that soil nutrients in this study area are mainly limited by nitrogen, with slow overall organic matter decomposition and a limited ability to mineralize and decompose phosphorus.

### Assessment of the benefits of salt reduction and soil modification in six microtopographies

The microtopographic modifications regulated surface roughness and improved soil water and salt conditions. Microtopographic variations trap plant seeds and promote the growth of *S. salsa*. Plant growth can further increase soil nutrients and reduce soil salinity. Therefore, a positive reciprocal feedback effect was established within the soil–plant system after microtopographic modification. However, the effect on plant growth and soil improvement varied greatly with the microtopographic modification model.

The six microtopographic modification patterns were divided into two categories: linear (e.g., strip-shaped, dense strip-shaped, and step-shaped) and curved (e.g., crescent-shaped, S-shaped, and pin-shaped). The evaluation of both soil physicochemical indexes and plant growth indexes revealed that the vegetation restoration effect of the three curved microtopographic modification patterns was greater than that of the three linear microtopographic modification patterns, and the soil improvement effect of the patterns with a linear shape was significantly greater than that of those with a curved shape. Among the microtopographic modification patterns, the soil improvement effect of the dense strip-shaped soil was significantly lower than that of the remaining five microtopographic modification models due to pit depth limitations. The dense strip-shaped soil performed best at reducing the soil salt content, which was significantly and positively correlated with the soil salt ion content, and there was no significant difference between the different soil layers. This result may be due to the regular shape and diverse orientation of the mound pits in this pattern, which are able to intercept plant seeds under different wind conditions. The strip-shaped pattern significantly increased the soil nutrient content. The reason for this finding may be the large area of the bottom of the strip-shaped pit, which can effectively intercept plant foliage and particulate organic matter carried by wind action. The regular shape and small bottom of the pin-shaped mound pits were conducive to the stability of microhabitats at the bottom of the pits, and they had a strong ability to reduce soil salinity and improve soil nutrients. The improvement in the S-shaped treatment was general. The reason for this finding may be that the irregular shape of the pits in this treatment does not lead to the formation of good microhabitats. The pit bottom area in the step-shaped treatment was large and surrounded by mound tops, making it difficult for seeds and nutrients to escape after being trapped; thus, this pattern promoted a uniform distribution of seeds at the bottom of the pit and an effective reduction in soil salinity. The number of pits set in the dense strip-shaped pattern was too high, and the pits were placed in a single direction, which was not conducive to the formation of airflow within the microtopography; furthermore, seeds were not easily trapped and were unevenly distributed and were therefore less effective for improving the soil. The assessment of microtopography transformation models contributed to the efficient and integrated use of high-salinity areas in the muddy coastal zone. This approach is important for the ecological protection and restoration of degraded coastal saline lands.

## Conclusion

By establishing microtopographic modifications, the roughness of the bare ground in the beach habitat is changed: seeds and surface litter are intercepted, soil physical and chemical properties are improved, and a staggered and uneven capture microarea is formed to promote seed landing and growth. Different microtopographic modification models showed differences in improving soil physicochemical properties and promoting plant growth, and the crescent-shaped pattern led to the most remarkable differences. Microtopographic modification could significantly improve soil nutrient contents, with significant increases in soil TC, TN, and TP contents. The most noticeable change in nutrient contents occurred in the treatment with the strip-shaped pattern, followed by that with the crescent-shaped pattern.

To improve soil water and salinity and promote plant growth, the crescent-shaped microtopographic pattern is most highly recommended, followed by the strip-shaped microtopographic pattern. To improve soil nutrient contents, the strip-shaped pattern is preferred, followed by the crescent-shaped pattern. It is recommended that when carrying out microtopographic transformation in the coastal beach of the Yellow River Delta, the crescent-shaped pattern should be considered first, followed by the strip-shaped pattern, and the dense strip-shaped pattern should not be used.

## Data availability statement

The original contributions presented in the study are included in the article/supplementary material. Further inquiries can be directed to the corresponding authors.

## Author contributions

KZ: Writing-original draft, Writing-review & editing, Investigation, Data curation. JX: Supervision, Funding acquisition. LS: Methodology, Investigation. FG: Investigation, Data curation. QC: Investigation, Data curation, XX: Investigation, Validation. MD: Investigation, Data curation. CL: Investigation, Data curation. All authors contributed to the article and approved the submitted version.
